# Evaluation of rK-39 strip test using urine for diagnosis of visceral leishmaniasis in an endemic area in Bangladesh

**DOI:** 10.1186/1756-3305-3-114

**Published:** 2010-11-26

**Authors:** Md Gulam Musawwir Khan, Mohammad Shafiul Alam, Milka Patracia Podder, Makoto Itoh, Kazi M Jamil, Rashidul Haque, Yukiko Wagatsuma

**Affiliations:** 1 International Centre for Diarrhoeal Disease Research, Bangladesh (ICDDR,B), 68 Shaheed Tajuddin Ahmed Sharani, Mohakhali, Dhaka 1212, Bangladesh; 2 Department of Zoology, University of Dhaka, Dhaka 1000, Bangladesh; 3 Department of Parasitology, Aichi Medical University School of Medicine, Nagakute, Aichi, Japan; 4 Department of Epidemiology, Graduate School of Comprehensive Human Sciences, University of Tsukuba, Ibaraki, Japan

## Abstract

Diagnosis of visceral leishmaniasis (VL) by demonstration of parasites in tissue smears obtained from bone marrow, spleen or lymph nodes is risky, painful, and difficult. The rK-39 strip test is widely used for the diagnosis of VL using blood/serum samples in endemic countries. The aim of the study was to evaluate the rK-39 strip test using urine sample as a non-invasive means for the diagnosis of VL. The rk-39 strip test was performed using urine from 100 suspected VL cases along with 25 disease control (malarial febrile cases) and 50 healthy control (from endemic and non-endemic areas). All the VL suspected cases were positive with the rK-39 strip test using serum. The sensitivity and specificity of the rK-39 strip test using urine samples was 95% and 93.3%, respectively, compared to serum based rK-39 test. The findings suggest that the urine based rK-39 test could be a practical and efficient tool for the diagnosis of VL patients in rural areas, particularly where resources are limited.

## Introduction

Visceral leishmaniasis (VL) is a serious public health problem in Bangladesh where 20 million people (18% of the total population) are at risk with a trend of rising incidence [1]. Diagnosis of VL still relies on clinical manifestations and microscopic confirmation of parasites from aspirates of lymph nodes, bone marrow, and the spleen. These invasive and painful techniques require skilled personnel and are difficult to implement in resource-limited settings. Several less-invasive serological tests, including indirect fluorescent antibody test (IFAT), enzyme-linked immunosorbent assay (ELISA), and an improved version of direct agglutination test (DAT) have been evaluated for the diagnosis of VL [2-4]. However, a rapid immune-chromatographic test (ICT) based on a recombinant 39-amino acid repeat antigen, conserved in the kinesin region of *Leishmania chagasi *and *Leishmania donovani *(rK-39 strip test), gained popularity for the field screening of kala-azar [5]. The detection of soluble antigen and antibody in urine of VL patients has been reported [6]. A urine-based ELISA method has also been developed to detect anti-*Leishmania donovani *immunoglobulin G (IgG) [7].

Recently, a low molecular weight, heat-stable, and carbohydrate based leishmanial antigen has also been detected in urine of VL patients [8]. A latex agglutination test (KAtex) based on antigen detection in urine of VL cases has been evaluated in different field studies; however, the test showed lower sensitivity in some studies [9,10]. So, the antibody detection tests especially DAT and rK-39 strip test, are still being extensively used in the field-screening of VL.

The study was conducted to determine the potential application of the rK-39 strip test for detecting anti-leishmanial antibody in urine for the preliminary diagnosis of VL infection compared with the serum-based rK-39 test to establish the value of the urine-based rapid test for the primary diagnosis of VL.

### Study area and population

In total, 100 suspected VL patients, who were positive with the serum based rK-39 strip test and had fever for at least two weeks, along with other clinical signs [11], were enrolled in this study from Trishal Upazila (sub-district) Health Complex (UHC) in Mymensingh district, which is one of the most endemic VL regions in Bangladesh. All the VL subjects were treated free of charge in the UHC as per the National Guideline and the WHO recommendations. To investigate cross-reaction with other diseases, twenty five (25) subjects with malaria were enrolled from a malaria-endemic area. To investigate subclinical infection, twenty five (25) healthy controls were enrolled who lived in the endemic area (Trishal) but did not have a past history of VL. Twenty five (25) healthy controls from non-endemic area were also enrolled for assessing the specificity of the urine rK-39 strip test. The serum rK-39 test was performed again in the field setting, a small laboratory in Trishal with that is near about 300 meters form UHC, whereas the urine rK-39 test was performed in the Parasitology Laboratory, ICDDR,B in Dhaka.

### Ethical approval

The Ethical Review Committee (ERC) and Research Review Committee (RRC) of ICDDR,B approved the study.

### Sample collection and methods

Finger- prick blood was taken in a capillary tube and transferred to a micro-tube (200 μm). Urine samples were also collected in a tube containing preservative (Na-azide) and stored at 4°C until transporting to the ICDDR,B. The blood sample was then centrifuged for separation of serum at the field laboratory (Trishal) where the rK-39 strip test (Kalaazar Detect™, InBios Inc., USA) was also performed as per the protocol of the manufacturer. Briefly, 1 drop of serum samples was applied to the base of nitro-cellulose strips impregnated with recombinant rK-39 antigen. After being air-dried, 3 drops of the test buffer (phosphate-buffered saline, plus bovine serum albumin) were added, and the strip was placed upright. The appearance of a lower red band (control) indicated the proper functioning of the test while the appearance of an upper red band indicated the presence of anti-rK-39 IgG, signifying a positive test. For urine assay, 3 drops of urine sample were applied directly to the strip without adding any test buffer. In both the cases the strip was observed after 10 minutes for the test band. A skilled laboratory technician performed the urine rK-39 strip test in the Parasitology Laboratory ICDDR,B who also monitored the serum rK-39 strip test at the field level.

### Data analysis

Sensitivity and specificity were computed along with 95% confidence interval (CI) using the Epi Info software (version 6.02; CDC, Atlanta, GA, USA). Data were also analyzed by 2×2 contingency tables using the SPSS software (version 10.0) for Windows (release 10.0.1, standard version, 1999; SPSS Inc., Chicago, USA), which enabled us to calculate the kappa coefficient or κ value. Reproducibility was assessed between the field rK-39 serum test and the urine rK-39 test in laboratory settings followed by Landis Koch [12] based on κ value.

## Results

The serum rK-39 strip test was positive in 100 enrolled VL subjects whereas all the healthy controls from the endemic area and non-endemic area and all the diseased controls (confirmed malaria subjects) were tested negative. The urine rK-39 strip test was positive in 95 out of the 100 VL subjects and in five out of 25 confirmed malaria patients who were tested negative in serum rK-39 strip test. However, the urine rK-39 was tested negative in all the healthy controls from the VL endemic and non-endemic areas. Thus, the sensitivity and specificity of urine rK-39 was found 95% (95% CI: 88.2-98.1) and 93.3% (95% CI: 84.5-97.5), respectively, considering the serum rK-39 test result as the gold standard (Table 1). Kappa coefficient (κ) for the urine rK-39 strip test was found 0.88.

**Table 1:** Comparison of urine and Serum based rK-39 strip test in the diagnosis of clinically suspected VL

Patient type	Serum rK-39 test		Urine rK-39 test	
	
	+ve	-ve	+ve	-ve
No. of VL case	100	0	95	5

No. of Malaria	0	25	5	20

No. of Non endemic healthy control	0	25	0	25

No. of VL Endemic healthy control	0	25	0	25

Total	100	75	100	75

Urine rK-39 strip sensitivity: 95% (95% CI: 88.2-98.1); specificity: 93.3% (95% CI: 84.5-97.5); kappa (κ): 0.88

## Discussion

According to the national guideline for the treatment of VL in Bangladesh, suspected kala-azar cases must be confirmed by a positive rK-39 or demonstration of parasite in the tissue (bone marrow/splenic puncture) or by PCR [11]. The ICT based rK-39 antibody test has been used widely in Bangladesh for the diagnosis of VL because of its high sensitivity and specificity [5].

According to the instruction of the manufacturer, this test is performed using serum or plasma for which collection of venous blood or figure- prick is necessary. But our study showed an excellent sensitivity and specificity level for the rK-39 dipstick test using a non-invasive procedure, i.e. urine samples. The sensitivity of the urine rK-39 strip test observed in our study (95%) corroborates the results of the urine-based DAT (90.7%) and urine ELISA (93.3%) [7]. The sensitivity and specificity of urine rK-39 in our study is within the acceptance level of the serum rK-39 strip test's sensitivity and specificity of specificity targeted (>90%) in the Indian subcontinent [13]. The urine-based rK-39 test has great advantages over the serum-based test because of ease of sample collection without causing any discomfort or pain to the subject. The non-invasive urine collection procedure minimizes the risk of blood-borne infections and facilitates the collection of samples from infants and children. Although, in our study a considerable number of positive test result with malaria (5/25) were noted in urine rK-39 test, but none was found in the healthy control subjects. We suspect this positive urine rK-39 strip test as false-positive as they were tested negative in the serum rk-39 test in the same malaria patient. This kind of false positivity might be raised due to the binding of unknown urinary components with the rK-39 antigen line in the test strip. According to Boelaert *et al. *[14], an ideal VL rapid diagnostic test should achieve a sensitivity level of ≥95% and a specificity level of ≥98% in both field and laboratory settings, and the test results should be interpreted in 30 minutes. The findings of our study showed that the non-invasive urine rK-39 strip test gave results in 10 minutes. The sensitivity of our test was satisfactory; however desired specificity was not achieved. The reproducibility of the urine rK-39 strip test was excellent (κ = 0.88) which corroborates with the reproducibility that had been assessed in a multi-centre evaluation of rK39 strip test with serum conducted in East Africa and the Indian subcontinent [13].

In our study the specificity has probably been overestimated because under real-life conditions there will be many malaria patients among the suspects to be tested. Moreover, half of the healthy controls were from a non-endemic area. The sensitivity may have been over-estimated in our study because the comparison was based on rK-39 sero-positive subjects. Another limitation of the rK-39 test is its variable sensitivity and specificity reported in different studies [15-18]. To overcome these limitations, further investigations are required to assess the performance of the urine rK-39 test in a larger field condition with a larger population size, including parasitological confirmed VL subjects to confirm our data.

## Conclusion

The urine rK-39 strip test would be a promising non-invasive *point-of-care *tool for the rapid screening of VL in remote rural areas where there is a high prevalence of VL. However, a large scale field evaluation of the urine rK-39 strip test is required before using it as a diagnostic tool for VL patients in different endemic areas. To the best of our knowledge, this is the first study on rK-39 strip test using urine samples.

## Competing interests

The authors declare that they have no competing interests.

## Authors' contributions

MGMK, MSA, MI, KMJ, RH, and YW have equally contributed in designing the study protocol. MSA was the PI of the project and was responsible for field set up and enrolling patients. MGMK, MSA, and MPP performed laboratory evaluation of the test. MGMK, MSA, and MPP drafted the manuscript. All authors edited, read, and approved the final manuscript.
